# Annotations

**Published:** 1899-06-17

**Authors:** 


### Annotations.

### The Medical Council and its "Pious Opinions."

It has long been a grievance, and it is no doubt a great hardship, that while the General Medical Council can by its own resolution, and without appeal, remove a practitioner from the Medical Register on the ground of his having been guilty of "infamous conduct in a professional respect," the Council has consistently refused to define "infamous conduct" in any but the most general terms. Each case, we are told, is decided on its own merits, and it is only by a study of the deeds of those who have been turned out that we can lay down for ourselves a line of conduct which shall lead to safety. This is forcibly brought home to us by a consideration of what happened at the recent meeting of the Council in regard to medical aid associations. If the Council would screw up its courage to take definite action in regard to this matter, and would say in so many words that men joining these associations as medical officers will be removed from the Register, such action would receive the hearty support of a large section of the profession; but it should give definite notice of its intention. What has happened is this. A resolution has been passed "that the Council strongly disapprove of medical practitioners associating themselves with medical aid associations which systematically practise canvassing and advertising for the purpose of procuring patients." Everything depends upon what this means. Is it to be taken as a notice that the Council is prepared to act, or is it merely a "pious opinion"? Sir John Batty Tuke, in discussing the resolution, said that "he would like to get some assurance that this was not the first stage in the construction of a new crime," and he added the common-sense remark that "they were certainly wasting their time if they were merely going to say that this or the other thing was very naughty." But he got no such assurance, and to ascertain the real meaning of the resolution we must be guided as far as may be by the speeches made while it was being discussed. These speeches then become of great importance. Now, the president said that the question which was referred to the committee whose report was under discussion had been raised by certain members of the profession "who practically asked them to adjudge those members of the profession who acted in connection with these associations to be guilty of infamous conduct in a professional respect." Then Mr. Brown, in seconding the resolution, said "it was the duty of the Council to interfere for the protection both of the profession and the public," and in regard to this remark it must be remembered that the Council can only interfere in one way. It is cognisant of only one crime—"infamous conduct"—and it can inflict but one penalty, namely, removal from the Register. Dr. McVail said "he would like to know whether the Council intended to take action in this matter . . . whether they intended to proceed against practitioners who did the thing complained of? If they did not intend to take action, then they should not pass this resolution, which would only be the expression of a pious opinion." But they passed the resolution all the same. Dr. Glover said that, "as to whether this motion was a piece of piety or a piece of resolution, so far as he was concerned it was a piece of intention and resolution, and he thought that the words of the motion were quite enough to give notice . . . that the Council would be prepared to use their disciplinary powers. . . . He assured the Council that, so far as he was concerned, he was in earnest in this matter." Under these circumstances, we can have no doubt that the meaning and intention of those who brought the resolution forward was clearly before the Council at the time the vote was taken, and when we add that the resolution was carried unanimously we think we may fairly say that unless the Council is prepared to stultify itself it will be bound to take action and to expel from the Register those who continue to act in defiance of its resolution. Whether it will do so is another matter, in regard to which we entertain many doubts. In any case, however, the profession labours under a great hardship and a serious grievance in that the Council has chosen so to word its resolution as to avoid stating in positive terms what it means to do. There are plenty of honourable men who will no doubt break off their connection with medical aid associations, and throw up a not inconsiderable portion of their income, purely and simply in deference to this expression of opinion by the Council, and if the Council should allow the appointments so vacated to be snapped up by less scrupulous persons the wicked will prosper and a great wrong will be done.

### Attractive Mortuaries.

The dedication last week of a mortuary chapel in connection with St. Augustine's, Stepney, draws attention to the sad lack which exists in our great cities of such necessary resting places for the dead. While we hear constant discussions in regard to their final disposal, the advocates of sepulture, interment, and cremation, each urging the advantages of their own pet schemes in terms which often, to say the least, appear tainted with exaggeration, it is curious how little is said about the provision of a proper and seemly resting place where the dead may lie until the time arrives for them to be carried to their last home. In a little village where the place of burial lies close to the dwellings of the living, and where perhaps the village pump may receive the drainage from the graveyard, the advantage of cremation would seem to be obvious enough. Yet those are just the localities in which it is never seriously proposed; whereas, in great towns it is apt to be made a question of the day—we had almost said a "burning question"—notwithstanding that intra mural burial has long since ceased to be, and that, so far as the health of the living is concerned, what is done with the corpse, after it is once removed to a far distant cemetery, becomes a

ANNOTATIONS—*continued.*

matter of indifference. When a body has once been removed to Woking, what can it matter to London whether it is burnt or buried? What is of interest, and what is of yearly growing importance as the population becomes more and more tightly packed, is the question. What is to be done with the corpse during the interval between death and its final disposition? Some may think it a misuse of terms when we say that we should make mortuaries "attractive," but the word exactly expresses what we mean. We would avoid, if possible, all exercise of compulsion in so delicate a matter as the removal of the dead, yet we cannot hide from ourselves that compulsion might become necessary for sanitary reasons; and we advocate the plan of making mortuaries attractive, partly because by so doing compulsion might thus be rendered unnecessary, and partly because, if compulsory removal should ever come, the resistance to it would thereby be lessened. Those who know best the condition of tenement life in London best know how urgent is this question of the early removal of the dead. To wrangle about cremation versus burial while corpses are left festering in crowded tenements is, indeed, straining at the gnat while the camel is swallowed whole.

### The Peace Congress.

Although the Peace Congress at the Hague originated in the proposal for universal disarmament made by the Czar, the chief discussions seem to have centred on proposals having for their aim the diminution of the horrors rather than the frequency of war, and England has received no small share of blame from the ultra-humanitarians for her refusal to undertake to give up the particular form of bullet which we have found it necessary to adopt in warfare with savage or semi-savage races. Under these circumstances it is well to insist that, destructive as the Dum-dum bullet undoubtedly is, it is nothing like so destructive as the old-fashioned uncoated bullets (to which no objection was ever raised) would be if it were possible to give them the velocity which modern bullets possess; and that, on the other hand, even a completely coated bullet will fly to pieces if it happens to hit a button or a bone, and will then inflict injuries exactly analogous with those produced by a soft-nosed projectile. The fact is, that it is a question of arms quite as much as of bullets, and that so long as we give to bullets the enormous amount of energy which is necessary for the great range which it is now thought necessary to attain, "setting up" and "disintegration" must take place under certain circumstances, whether they are soft-nosed or not. But, after all, if we want to see really ghastly injuries, let us turn to the weapons by which the real fighting is done in a battle between civilised nations. Shells are far worse than bullets.

### The House of Commons and the Army Medical Officers.

The answer given by Mr. Balfour to Dr. Farquharson in the House of Commons, in regard to the absence of the names of medical officers among those to whom it was proposed that the thanks of the House be given for their services in the Soudan campaign, is one which, if it is to be taken as a precedent, means that under no circumstances are officers of the Royal Army Medical Corps to receive such a distinction. "We have gone," he said, "on the principle that you should not go below the rank of general of brigade or some rank equivalent to that." The principle is intelligible and is no doubt pleasing to the purely fighting branches of the services. At the same time those who look at matters from other points of view will see at once that some of those included in the list, highly placed as they were, had merely to carry out the orders given to them, whereas those responsible for the medical conduct of the campaign had laid upon them the duty of initiating and carrying out a whole series of measures on the proper conduct of which the success of the expedition to a large extent depended. People who look at the matter in this light will probably consider that, if any are to be picked out by name for special honour, it would be more fitting to select the heads of important departments than to go down to a certain level of rank in one branch and there draw the line.

### The Metropolitan Provident Medical Association.

At a meeting which was lately held in favour of the Metropolitan Provident Medical Association many pleasant things were said about the provident system as applied to the provision of medical attendance for the poor with much of which we heartily agree; but it is no use covering up difficulties with pleasant speeches, and it must be obvious to everyone that the institution in question is by no means succeeding as it was expected to succeed when it was first launched, and we can hardly doubt that its difficulty is that no one can say whether it is a charity or a business undertaking. One would think that an institution partly supported by subscriptions, advocated at what we suppose we may call "drawing-room meetings," and receiving grants from the Hospital Sunday Fund, &c., might fairly be called a charity. That, however, is not the view accepted by those who receive its benefits. It is maintained that its provident members pay for what they get, and are beholden to no man, and if that is so we not unnaturally inquire how much they pay. According to a statement made at the meeting the payments from "more than 30,000 persons" amounted to "over £5,300 a year." This works out to a good deal under a penny per week per person, and we fancy we shall not be far out if we say that a little less than a half-penny comes to the doctors who do the work. Now if the doctors are content to see all classes of patients for fees like this, as a mere business undertaking, we have nothing more to say. Medical advice is cheaper than we thought, and there is an end of the matter. But if the medical men accept these small fees because the whole affair is a charity arranged for the benefit of the poor, these dispensaries ought to be worked under as strict a wage limit as those that are purely eleemosynary. If the Metropolitan Provident Medical Association fails of the entire success which, considering its object, it ought to obtain, it is probably because it falls between two stools. The claim put forward that the provident members pay for all they get hardly tends to conciliate the *amour propre* of the medical profession revelling in their half-penny a week, while the undoubted tinge of patronage and charity which attaches to these dispensaries alienates the independent working man. The working man is ready enough to pay his money. He is also prepared on occasion, and for a consideration, to touch his hat. But to touch his hat and pay as well is more than he can stand.

